# Lymphaticovenular anastomosis for Klippel-Trenaunay-Weber syndrome

**DOI:** 10.1016/j.ijscr.2019.04.023

**Published:** 2019-04-16

**Authors:** Satoshi Onoda, Sho Komagoe

**Affiliations:** a Department of Plastic and Reconstructive Surgery, Kagawa Rosai Hospital, Kagawa 763-8502, Japan; b Department of Plastic and Reconstructive Surgery, Okayama Saiseikai Genaral Hospital, Okayama, Japan

**Keywords:** Lymphaticovenular anastomosis, Klippel-Trenaunay-Weber syndrome, Vascular malformations, Cellulitis

## Abstract

**Introduction:**

Since we performed lymphaticovenular anastomosis (LVA) for Klippel-Trenaunay-Weber syndrome and obtained relatively good results, we report the adaptation, effect of treatment, and mechanism of LVA.

**Presentation of case:**

Case is a 28-year-old man with an increase of the circumference and pigment changes on the whole right leg and gluteal region from the time he was born. He exhibited signs of right leg cellulitis with a fever about 40° at frequency of the degree once a month. We planned to perform LVA of the right leg to prevent cellulitis of the lower limbs.

**Discussion:**

This case presented with repeated, severe cellulitis occurring once a month prior to treatment. This symptom greatly reduced the quality of life of the patient. Postoperatively, he lived his daily life with no particular limits, and significant improvement quality of life was due to LVA. Therefore, it is inferred that the protective efficacy of inflammation was obtained by anastomosing lymphatics and the vein around the skin lesions and a return current of the lymph flow was promoted.

**Conclusion:**

The possibility that LVA could become an excellent therapy for similar cases is suggested.

## Highlights

• To the best of our knowledge, this is the first report on lymphaticovenular anastomosis (LVA) for the Klippel-Trenaunay-Weber syndrome.
• We performed seven LVA procedures for the right lower limb with the intention of preventing recurrent bouts of cellulitis.
• The patient has been cellulitis-free for 1 year post-operatively, having previously had such infection every other month.
• We suggest that LVA may be effective for the management of similar cases.

## 1 Introduction

Klippel-Trenaunay-Weber syndrome is a vascular, lymphatic malformation disease spread over the whole or distal segments of arms or lower limbs and laterality occurred in appendicular size and form. As a vascular malformation, it is complicated by capillary malformation, venous malformation, arterio-venous malformation, lymphatic malformation, and these vascular malformations are mixed in most cases. Additionally, patients with these vascular malformations often present with symptoms, such as lymphatic swelling, due to lymphatic dysfunction and cellulitis, etc. These symptoms are often aggravated with aging or growth. Since we performed lymphaticovenular anastomosis (LVA) for Klippel-Trenaunay-Weber syndrome and obtained relatively good results, we report the adaptation, effect of treatment, and mechanism of LVA. This work has been reported in line with the SCARE criteria. [1]

## 2 Case

**Fig. 1:**
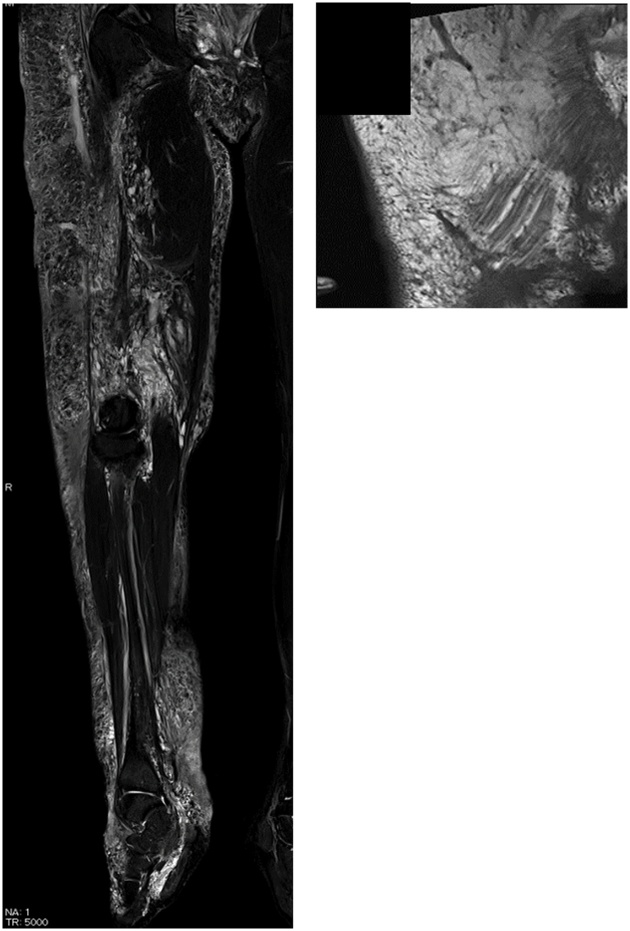
Vascular malformation was detected in the whole right leg and buttocks via MRI at the initial diagnosis. The mixed vascular malformations were noted in a thigh and the buttocks with skin lesions in particular.

**Fig. 2:**
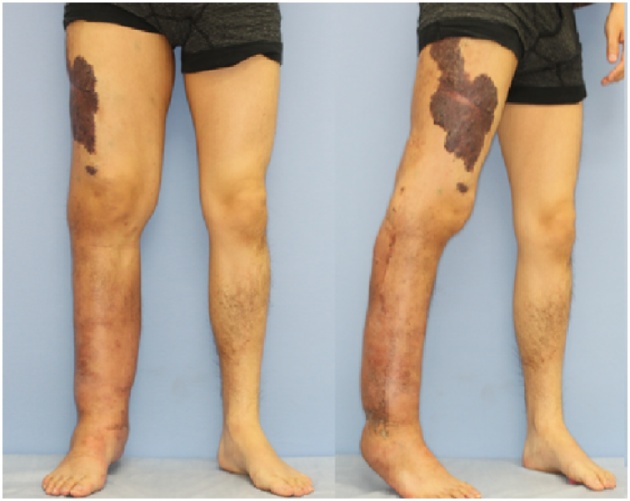
The photographs of the lower limbs at the initial diagnosis.

**Fig. 3:**
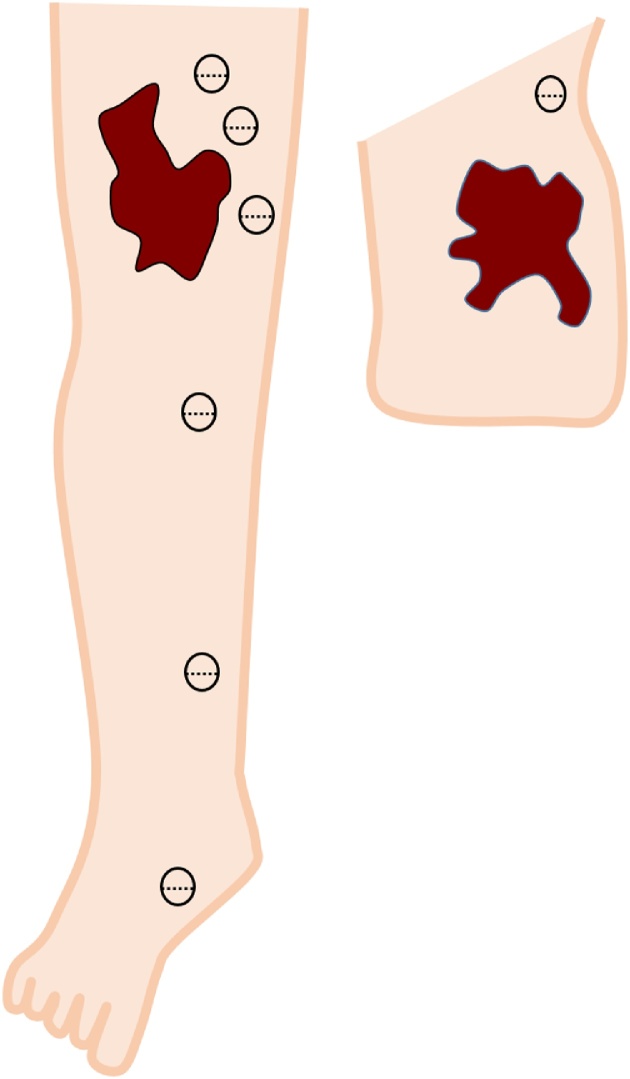
The site of lymphaticovenular anastomosis. We anastomosed seven areas, from the ankle to the buttocks.

**Fig. 4:**
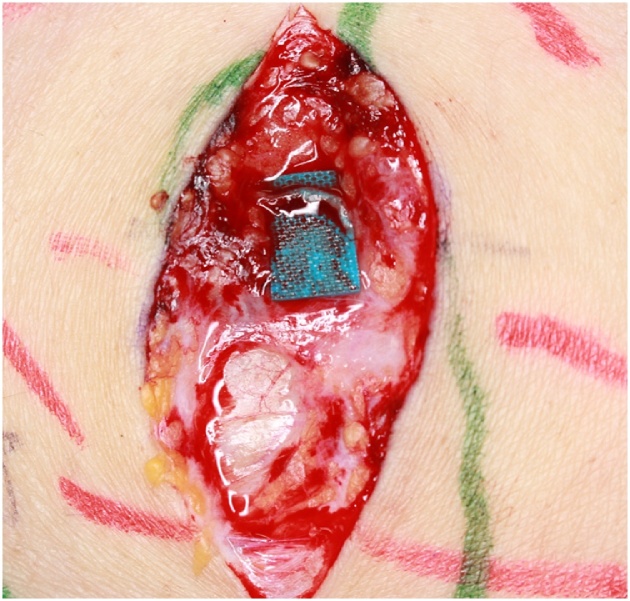
Anastomosis in the groin site. We performed end to end anastomosis in a 0.55 mm vein and a 0.7 mm lymphatic duct.

A 28-year-old man was admitted due to an increase of the circumference and pigment changes on the whole right leg and gluteal region from the time he was born. Segmental resection was performed multiple times during childhood at another hospital. Pressure therapy with normal stockings was performed intermittently, but his symptoms gradually worsened and our hospital was subsequently consulted. At consultation, swelling was detected in the whole right leg and showed a difference in circumference between the left and right legs (Fig. 1). In addition, he exhibited signs of right leg cellulitis with a fever about 40° at frequency of the degree once a month. In addition, pain of NSR (Numerical Rating Scale) 4–5 degree was detected during inflammation. The measurements (cm) for the right lower extremity diameter at initial diagnosis were as follows: dorsalis pedis, 22.5; ankle, 28.6; 10 cm below the knee joint, 35.8; knee joint, 42.0; and 10 cm above the knee joint, 45.0. He started wearing elastic stockings for lymphedema after consultation with our department and symptoms resolved at once. However, because he developed cellulitis again, we planned to perform LVA of the right leg to prevent cellulitis of the lower limbs. We conducted lymph flow evaluation by indocyanine green angiography preoperatively. At first, indocyanine green was injected on the tip of the foot as part of normal indocyanine green angiography, but the dye almost did not move from injection site. Next, we injected indocyanine green around skin lesions on the femoral and gluteal areas (Fig. 2). We identified voluminous, significant lymph, which flowed out from skin lesions. We performed 7 lymphaticovenular anastomosis at the femoral region, groin region, the calf, ankle joint, and the buttocks (Fig. 3, Fig. 4). As for the lymph that entered the anastomosis, the smallest diameter of the anastomosis 0.35 mm and the maximal diameter was 0.8 mm. In particular, we anastomosed the lymphatic duct and vein near the border of the lesion from normal tissue in the area of the femoral and gluteal skin lesions. The operative time was 4 h and 28 min.

The measurements (cm) for the right lower extremity diameter at one week after operation were as follows: dorsalis pedis, 21.5; ankle, 27.5; 10 cm below the knee joint, 33.5; knee joint, 42.0; and 10 cm above the knee joint, 46.0. Therefore, slight improvement in the condition was observed. The operation caused infection to develop in the surgical suture in the ankle, postoperatively, but symptoms cleared by removing the thread. The patient has been cellulitis-free for 1 year post-operatively and has been able to live his daily life without any problems. He uses elastic stockings intermittently. The patient has agreed to the publication of this paper.

## 3 Discussion

There is no radical therapy for the pathologic increase in growth as treatment for Klippel-Trenaunay-Weber syndrome. Surgical correction surgery (epiphyseal line growth suppression technique, bone elongation) or harnesses assisting the lower limbs, are performed for leg-length difference. Additionally, debulking surgery, such as lesion resection, may be performed for addressing leg-length discrepancy. For the vascular malformation, therapeutic strategies vary according to symptoms, the characteristics of the vessel, and the frequency of cellulitis. Generally, pressure therapy with elastic stockings, tumor resection, sclerotherapy, and embolization are performed, but all these therapies are considered as palliative treatment. There is no effective therapy for patients that suffer from repeat cellulitis with lymphatic malformation and appendicular edema, in particular. LVA is performed as surgical treatment for secondary lymphedema primarily, and protective efficacy of the cellulitis [2,3] and edema reduction was reported [[4], [5], [6]]. Also, at the animal experiment level, one end of the postoperative change is elucidated. [7,8] Additionally, the protective efficacy of lymphedema through LVA was reported [9].　

This case presented with repeated, severe cellulitis occurring once a month prior to treatment. Visiting the hospital and hospitalizations were required each time. This symptom greatly reduced the quality of life of the patient. Postoperatively, he lived his daily life with no particular limits, and significant improvement quality of life was due to LVA. Inflammation occurred from the femoral and gluteal skin lesions, and inflammation was spread in the whole lower limbs preoperatively. Therefore, it is inferred that the protective efficacy of inflammation was obtained by anastomosing lymphatics and the vein around the skin lesions and a return current of the lymph flow was promoted.

For the Klippel-Trenaunay-Weber syndrome, many cases present with lesions occurring in the whole lower limbs, similar to this case. It is thought that the protective efficacy of cellulitis might be obtained by performing improvement of the lymph flow using LVA around the densest vascular lesion, causing inflammation such as skin lesions. However, there are many points that are not understood, including the optimal site of LVA and other matters that require attention for cases such as this. It is necessary to examine the improvement effect against edema or the protective efficacy of cellulitis by performing LVA in more cases in the future.

## 4 Conclusion

We performed LVA for a case of Klippel-Trenaunay-Weber syndrome, which presented with frequent cellulitis and showed excellent effect of treatment. The possibility that LVA could become an excellent therapy for similar cases is suggested.

## Conflicts of interest

The authors have no financial interest to declare in relation to the content of this article.

## Funding

This work was supported by JSPS KAKENHI Grant Number 17K17016.

## Ethical approval

Ethical approval has been exempted by our institution in case reports.

## Consent

Informed consent was obtained from the patient for the publication of this case report and accompanying images.

## Author contribution

Satoshi Onoda: performance of the treatment.

Sho Komagoe: performance of the treatment.

## Registration of research studies

NA.

## Guarantor

Satoshi Onoda and Sho Komagoe.

## Provenance and peer review

Not commissioned, externally peer-reviewed.
